# Short-Term Postoperative Outcomes after Resective Colorectal Surgery in Elderly vs. Nonelderly Patients: A Single Centre Retrospective Analysis

**DOI:** 10.3390/cancers16193358

**Published:** 2024-09-30

**Authors:** Vincenzo Tondolo, Federica Marzi, Luca Emanuele Amodio, Gianluca Rizzo

**Affiliations:** Digestive and Colo-Rectal Surgery Unit, Ospedale Isola Tiberina Gemelli Isola, 00186 Rome, Italy; vincenzo.tondolo@fbf-isola.it (V.T.); federica.marzi01@icatt.it (F.M.); lucaemanuele.amodio01@icatt.it (L.E.A.)

**Keywords:** colorectal cancer, elderly, colorectal surgery, postoperative morbidity

## Abstract

**Simple Summary:**

According to increasing life expectancy, an increasing number of elderly people need colorectal resective surgery to treat colorectal disease, especially colorectal cancer. In the last years the surgical techniques were deeply modified, also in colorectal surgery, with progressively higher rate of minimally invasive surgery and with a progressively higher rate of adhesion to fast-track protocols. The introduction of these new factors significantly modified the post-operative outcomes after colorectal resective surgery, but in elderly patients these advantages were less evident in literature. The aim of this study was to evaluate and quantify the impact of advanced age on short-term postoperative outcomes.

**Abstract:**

**Background/Objectives:** Life expectancy for people in their 60s is 24.3 years in high-income countries. Health systems face the burden of disease in the elderly population and must assess the impact of treatments such as major surgery. The aim of this study is to quantify the impact of advanced age on short-term postoperative outcomes after resective colorectal surgery (RCRS). **Methods**: All patients who underwent RCRS at our institution between July 2022 and November 2023 were entered into a database. Preoperative, perioperative, and early (within 30 days) postoperative data were recorded. Patients were categorized into a young group (under 75 years, YG) and an elderly group (over 75 years, EG). A retrospective comparative analysis of postoperative outcomes was performed between the two groups; postoperative complications were graded according to the Clavien classification. **Results**: Fifty-three and ninety-five patients were in the EG and YG, respectively. Indications for RCRS was cancer in 83% of EG patients and 61.1% of YG patients (*p* = 0.006), and the clinical presentation, localization, and rate of neoadjuvant treatment in oncological patients were comparable. Another indication for RCRS was complicated diverticular disease (17% of EG patients and 38.9% of YG patients; *p* = 0.006). With respect to the baseline characteristics, the ASA and CCI scores were worse in the EG (*p* = 0.001). No significant differences in the surgical approach, mini-invasive approach, conversion rate, definitive stoma creation, or number of harvested lymph nodes were found between the two groups. Overall, EG reported a higher relative risk (RR) of short-term postoperative complications (1.64, CI: 1.03–2.63), but no significant differences were found in terms of grade ≥3 complications (RR: 0.9, CI: 0.23–3.44). In the EG, a higher risk of ICU admission (RR:2.69, CI: 1.5–4.8) and a one-day longer postoperative hospital stay (6 vs. 5 days) were reported. **Conclusions**: Advanced age does not seem to contraindicate RCRS, especially in colorectal cancer patients. The impact of elderly age on short-term outcomes seems to be minimal and acceptable.

## 1. Introduction

The population in high- and upper–middle-income countries is progressively aging. The proportion of elderly individuals (65 years and over) in the EU-27 total population is projected to increase from 20.3% (90.5 million) at the beginning of 2019 to 31.3% (130.2 million) by 2100 throughout all 31 European countries. In Italy, data collected in 2018 showed that life expectancy at birth is 83.4 years, with a healthy life expectancy of 66.8 [1]. Elderly patients are often affected by several comorbidities with consequently decreased physiological reserves, increasing their vulnerability to adverse events and increasing the complexity of clinical management and therapeutic decisions. These factors could be challenging in the setting of surgery, which often involves general anesthesia and represents a stress that could be rapidly fatal in patients with low physiological reserves [2].

Colorectal cancer and diverticular disease represent the most frequent indications for colorectal surgical resection in elderly patients. Colorectal cancer is the second most common cause of cancer-related deaths in men and the third most common cause in women, and its incidence significantly increases between the 7th and 8th decades [3,4,5]. Analogous to that of colorectal cancer, the prevalence of diverticular disease significantly increases with age, from 10% in young patients (<40 years) to 50 to 70% in those over 80. With increasing age, the rate of patients presenting with complicated diverticular disease requiring surgical treatment increases, carrying a consequently high rates of postoperative morbidity and mortality, especially in elderly patients and emergency settings [6,7].

Over the last 30 years, the diffusion of novel surgical techniques (such as minimally invasive approaches) and ERAS protocols has significantly improved the perioperative outcomes of patients undergoing colorectal surgery [8]. Although elderly patients are usually excluded from clinical studies, evidence published in the literature confirms the feasibility of minimally invasive and ERAS approaches in elderly patients, including those who need colorectal surgery both for oncological and benign diseases, with the same advantages recorded in younger patients [8,9]. In addition to perioperative strategies, the key point in managing the elderly population is tailoring treatment by assessing the frailty of the patient throughout a comprehensive geriatric evaluation that can better define the physiological reserve and expectancy of life [10,11].

The aim of this study was to evaluate the feasibility of colorectal resection surgery (for both benign and malignant diseases) in elderly patients (over 75 years) in terms of short-term postoperative outcomes and the feasibility of novel techniques.

## 2. Materials and Methods

### 2.1. Study Design and Population

All patients who underwent elective colorectal resection at Isola Tiberina Hospital-Gemelli Isola in Rome from July 2022 to July 2023 were retrospectively enrolled in the study. The study population was categorized into two groups: the elderly group (EG), comprising patients aged 75 years or older, and the younger group (YG), comprising patients under 75 years. Moreover, patients were categorized into two groups according to indications for colorectal surgery: benign disease (complicated diverticular disease) or malignant disease (colorectal cancer).

The preoperative work-up varied according to the indications for surgical colorectal resection. For benign disease, patients underwent colonoscopy and abdominal CT, and surgical indications were determined by the surgeon on the basis of clinical presentation and evidence of signs of complicated disease (stenosis, recurrent flares of diverticulitis, sequelae of perforation/chronic abscesses and/or fistula). For malignant disease, all cases were preoperatively discussed with a dedicated multidisciplinary tumor board (MDTB) for colon and rectal diseases to assess tumor stage and treatment strategy. Specifically, in cases of colon cancer, clinical staging, and resectability were assessed based on a preoperative whole-body computed tomography (CT) scan, and indications followed the most recent guidelines for colon cancer treatment [12]. In rectal cancer, tumor staging was completed by adding pelvic magnetic resonance imaging (MRI) to evaluate the tumor location (intraperitoneal or extraperitoneal rectal cancer) and locoregional staging (early or locally advanced). Indications for upfront surgery or chemoradiotherapy were based on the most recent National Comprehensive Cancer Network (NCCN) guidelines [13], multidisciplinary discussion, and the consideration of individual patient’s physical status. An anesthesiologist’s preoperative work-up was performed according to the American Society of Anesthesiologists (ASA) physical status classification system.

Perioperative data were collected from a prospectively maintained database. The clinical and demographic characteristics included age, sex, BMI, Charlson Comorbidity Index score, and ASA score. The following perioperative features were also collected: indications for colorectal surgical resection (benign or malignant disease), tumor location, preoperative clinical stage, preoperative neoadjuvant chemotherapy and/or radiotherapy, the type of surgical approach (laparoscopic or open), rate of conversion, intraoperative complications, admission to the intensive care unit (ICU) after surgery, length of stay in the ICU, day of resuming oral intake after surgery, short-term (within 30 days) postoperative complications (classified according to the Clavien–Dindo classification), type of complication, and short-term postoperative mortality. In patients with rectal cancer, the site of the tumor was further classified as the lower rectum (from the internal anal orifice (i.a.o.) to 5 cm from the i.a.o.), middle rectum (5 to 10 cm from the i.a.o.) or high rectum (10 to –15 cm from the i.a.o.). For oncological patients, additional data collected included the number of harvested lymph nodes, the number of metastatic nodes, and the pTNM stage according to the AJCC 8th Edition [14].

### 2.2. Operative Technique

All colorectal surgical resections were performed under general anesthesia via multimodal analgesic techniques, including preoperative spinal anesthesia, if feasible. In all cases, minimally invasive approaches were adopted whenever feasible.

In malignant disease, the extent of surgical colonic or colorectal resection varied according to the tumor location. All colonic patients eligible for radical surgery underwent surgery involving high-tie ligation of the main vessels, extensive lymph node dissection around the origin of the vessels, and complete mesocolic excision (CME). All rectal cancer patients underwent radical resection according to tumor location: intraperitoneal tumors underwent anterior resection, partial mesorectal excision (PME), and colorectal anastomosis, while extraperitoneal tumors underwent anterior resection with total mesocolic excision (TME) and coloanal anastomosis, if feasible. For patients with low rectal cancer lacking a cancer-free resection margin from the anal canal, abdominoperineal resection with TME was performed.

In diverticular disease, a colorectal resection, including all the colonic tracts involved in complicated diverticular disease, with mechanical end-to-end colorectal anastomosis was performed. In all cases of left colon or rectal resection, despite the etiology (tumor or benign disease), IMA ligation and splenic flexure mobilization were used to assure tension-free anastomosis and provide definitive treatment in cases of incidental findings positive for malignancy. An air leakage test was systematically conducted after performing a colorectal anastomosis via a disposable proctoscope; a positive air leakage test was defined by the presence of bubbles in the lavage fluid within the pelvis. A protective stoma was created after intestinal anastomosis in all previously irradiated patients and those at high risk of postoperative anastomotic leakage. A drain was always placed near the anastomosis or in the pelvis.

### 2.3. Postoperative Management

Patients were transferred to the surgical ward or ICU according to the anesthetist’s clinical judgment. The first mobilization after surgery and urinary catheter removal were usually performed on the first postoperative day. An oral diet was progressively started from the first postoperative day. The abdominal drain was removed at the resumption of intestinal function (gas or stool canalization). If no postoperative complications occurred within the fourth or fifth postoperative day, patients were discharged.

### 2.4. Study Outcomes

The primary endpoint was a comparative analysis of the rate and severity of short-term postoperative complications (within 30 days) between the EG and YG. The secondary endpoint was a retrospective analysis of the impact of advanced age on operative strategies (rate of mini-invasive approach, extension of lymphadenectomy, rate of temporary and definitive stoma, and rate of temporary stoma closure).

### 2.5. Statistical Analysis

Continuous data are reported as medians (IQR, 25–75th interquartile), whereas categorical variables are expressed as numbers and percentages. Comparative analysis on baseline characteristics was performed via Fisher’s test or X^2^ test (for categorical variables) and Mann–Whitney *U* tests or *t*-tests (depending on the data distribution curve). A *p*-value ≤ 0.05 was considered statistically significant. Outcomes variables, except “length of stay”, are described as relative risks in consideration of the small sample size and low statistical power. Analyses were performed via Wizard (version 1.9.49) for MacOS.3.

This study represents a satellite study of the MINDS FOR FRAILITY.01 23 (protocol approved by the Institutional Review Board on 31 October 2023), and the retrospective analysis of data started after the approval of the Institutional Review Board.

## 3. Results

From July 2022 to July 2023, 148 patients underwent elective colorectal surgical resection: 53 patients in the EG and 95 in the YG. Table 1 shows the demographic characteristics of the patients included in the study. In the EG, the median age was 81 years, and 35 patients (66%) were over 80 years old. The median age in the YG was 62 years (35–74). The EG had an ASA score that was significantly higher than that of the YG (*p* = 0.002), and the Charlson Comorbidity Index was seven in the EG, which was significantly higher than that in the YG (3; *p* = 1.03 × 10^−9^).

With respect to indications for surgery, 44 patients (83%) in the EG had colorectal cancer, and 9 patients (17%) had diverticular disease; this distribution of indications for surgery was significantly different from that in the YG, where 58 patients (61.1%) had colorectal cancer, and 37 patients had diverticular disease (*p* = 0.006).

Table 2 and Table 3 present the oncological and clinical features of patients affected by colorectal cancer and diverticular disease, respectively.

In the setting of colorectal cancer patients. EG patients showed a 1.4 RR of a complicated pattern and were more frequently affected by right-sided colon cancer than YG patients (54.5% vs. 40.7%). No major differences in the pathological TNM stage were found between the two groups (Table 2).

The median number of harvested nodes was not significantly different between the EG and YG (20 vs. 22; *p* = 0.662).

With respect to diverticular disease, the percentage of patients who underwent surgery for complicated diverticular disease was significantly greater in the YG (37.9% vs. 16.9%; *p* < 0.05); moreover, the indications for surgery for diverticular disease were significantly different between the YG and EG, with a higher percentage of YG patients who underwent surgery for recurrent acute episodes of diverticulitis and a higher percentage of EG patients who underwent surgery for stenosis (*p* = 0.017).

The perioperative data and postoperative outcomes are summarized in Table 4. The rate of minimally invasive surgery was similar in both groups (92.5% in EG vs. 94.7% in YG), and the rate of conversion in the two groups was similar (14.3% in EG vs. 7.7%). The RR for definitive stoma creation was greater in the EG (RR 2.39, CI 0.56–10.28), while the RR for a temporary stoma was greater in the YG. All temporary stomas in the EG were closed within 2 months of their creation.

With respect to postoperative outcomes, a greater percentage of EG patients were admitted to the ICU during the immediate postoperative period (RR of 2.69 1.5–4.84), and the median length of stay in the ICU was 1 day in both groups. Twenty-five patients (47.2%) in the EG experienced postoperative complications, a rate significantly higher than the rate recorded in the YG (30.5%); however, no exceeding RR was found in the EG in terms of grade ≥3 complications (RR: 0.9, CI 0.23–3.44) A per-group analysis of the types of postoperative complications that occurred in both groups revealed a higher risk for EG in terms of paralytic ileus, acute urinary retention, and wound infection, which were greater in the EG than in the YG (18.8% vs. 2%; 11.3% vs. 3.1%; 11.3% vs. 6.25%). The difference between the EG and YG in terms of the overall rate of postoperative complications, even if no differences were found for major (grade ≥ 3) complications, also influenced the length of postoperative hospital stay, which was significantly longer in the EG than in the YG (median of 6 days in the EG vs. 5 days in the YG; *p* = 0.014).

## 4. Discussion

Life expectancy in Western countries is progressively increasing, resulting in a progressively increasing percentage of elderly people [1,2]. Thus, the number of elderly patients who need surgery has increased, increasing the amount of related critical issues [15]. Those issues are mostly adverse perioperative outcomes (postoperative outcomes as early postoperative mortality) related to the decreased physiological reserve and the several comorbidities that affect up to 75% of octogenarians [16].

In our one-year personal series, the percentage of elderly patients, defined as those over 75 years of age, who underwent colorectal surgery was 35.8%, highlighting the increasing median age of the population that require surgery at our institution. Similar to data reported in the literature, in our series, the EG was affected by a higher rate of comorbidity and anesthesiologic risk, as demonstrated by the significantly higher CCI and ASA scores in the EG.

In our series, patients underwent surgery for colorectal cancer and for complicated diverticular disease.

Colorectal cancer is the second most common cause of cancer-related deaths in men and the third most common cause of deaths in women. Its incidence significantly increases between the 7th and the 8th decades, as confirmed in the EU-27 report of 341,419 colorectal cancer patients (2020), in which patients over 70 years of age represented 57.36% of the cohort [4]. Similarly, the prevalence of diverticular disease significantly increases with age, from 10% in young patients (<40 years) to 50–70% in those over 80 years of age. In the clinical history of diverticular disease, increasing age increases the risk of surgery, which is indicated if a complicated disease occurs in both elective and emergency settings.

Despite similar age-related trends in incidence, the distributions of the indication for colorectal surgery in our series were significantly different between the YG and EG, with a significant predominance of colorectal cancer in the EG. This reason lies in the different histories of the diseases. On the one hand, for diverticular disease, recent studies suggest that the risk of perforation is highest at the first episode (5–25%) but decreases progressively with the number of subsequent attacks, requiring a more tailored indication for surgical treatment after coming through the first episode with conservative management [17]. Nonetheless, in recurrent symptomatic patients, elective sigmoidectomy was shown to improve quality of life, but the indication for surgery should be balanced with the perioperative risks [18,19]. This seems to justify why, in our series, the EG underwent elective surgery mostly due to critical stenosis or abscesses secondary to diverticula perforation, while in the YG, the most frequent indication was recurrent episodes of diverticulitis. On the other hand, in the context of colorectal cancer, surgical treatment is the cornerstone of the curative strategy to prevent or delay the local and systemic spread of the tumor. In our series, the EG tended to have a greater rate of complicated colorectal cancer (by stenosis or anemia) than the YG. This evidence seems to be coherent with other studies reporting a higher incidence of advanced tumors in elderly patients. [20]. Elderly colorectal cancer patients are reported to be more frequently affected by T4-stage tumors at the moment of diagnosis and to have a significantly higher rate of emergency surgery than young patients [15]. This significantly higher rate of emergency treatment in elderly patients with colorectal cancer was also reported by the large SEER-Medicare Database (31.574% of patients were over 80 between 1992 and 2005), with a rate of emergency admission of 46% in elderly patients, and consequently, a negative effect on 1-year survival [21], suggests the need for evolving colorectal cancer screening strategies in patients aged 75–84 years on the basis of individual health status, life expectancy, and screening history [22,23].

Focusing on the feasibility of surgical strategies, we described the number of harvested lymph nodes as a key parameter in assessing the radical intent of oncological colorectal surgery, demonstrating comparable accuracy in terms of lymph node dissection between the two groups. These results were achieved with minimally invasive surgical techniques with comparable rates (90%) in both groups despite the challenges characterizing the EG (greater anesthesiologic risk during patient positioning and pneumoperitoneum induction; locally advanced cancers; left colon diverticulitis with chronically stenotic bowel or abscesses). Minimally invasive surgery, often combined with fast-track or ERAS protocols, plays a well-known crucial role in enhancing postoperative outcomes following colorectal surgery [24,25]. In elderly patients, the feasibility and advantages of minimally invasive approaches have not been clearly demonstrated due to the fear of their hemodynamic impact on elderly physiology and functional reserve as well as emergency presentation. In the multicenter comparative analysis by Hinoi et al. between laparoscopic and open colorectal surgery for colorectal cancer in patients over 80, the mini-invasive approach was associated with a significantly lower rate of postoperative morbidities, lower blood loss, and a lower time to oral intake than open surgery [26]. This evidence was also confirmed by a meta-analysis of 24 studies, which compared clinical and survival outcomes between laparoscopic and open surgery in elderly colorectal cancer patients (over 65 years of age); in this analysis, the MIS group had a lower risk of mortality (within 3 months postoperatively) and a lower rate of postoperative morbidity (in terms of blood loss, length of hospital stay and time of return of bowel movements) than the open surgery group. Moreover, no differences in long-term outcomes were found between the two groups [27]. Despite MIS, advanced age still represents an independent risk factor for increased postoperative complications and mortality in colorectal surgery patients. Turri et al., in a retrospective analysis of 1482 colorectal cancer patients undergoing surgery, reported a double incidence of postoperative complications in patients with comorbidities (32.8% vs. 15.1%, *p* = 0.002) and an overall survival strongly dependent on age, with older people dying at a higher rate from competing causes than from cancer-related treatments compared with patients of younger ages [16]. Consistent with what has been reported previously in the literature, we found RR in terms of major postoperative complications (grade ≥ 3 according to the Clavien classification) to be comparable between the two groups, but a higher RR of minor (grade 1–2) complications and ICU admission in the EG, leading to a higher postoperative length of hospital staying. Data on long-term outcomes were obtained as survival was not an object of this study. Data obtained in this study, despite the low statistical power related to the small sample, carried favorable measures of relative risk that suggested a feasible and safe surgical intervention in the elderly population. At the same time, it induced some relevant considerations related to this frail category of the population. Surgical intervention, even when uncomplicated, can significantly impact the functional reserves of elderly patients, exacerbating pre-existing dysfunctions and accelerating systemic decompensation. The remaining challenges include optimizing postoperative risk in patients with limited life expectancy and multiple comorbidities, as well as stratifying elderly patients to identify those who could benefit from surgical intervention with long-term survival. Several studies have reported similar disease-specific survival rates between elderly and younger colorectal cancer patients but higher overall mortality rates in elderly groups [16,28]. Elderly colorectal patients require a multidisciplinary approach to establish their frailty, stratify their perioperative risk, and optimize their health status, which can reduce the risk of postoperative complications. The commonly used preoperative risk assessment scores (ASA Physical Status Classification System and ACS NSQIP Surgical Risk Calculator (including functional parameters, age, and procedure type) often overlook frailty [29,30]. Frailty, a critical factor in elderly patients characterized by progressive decline and heightened vulnerability, is established not only by age but seems to significantly influence the perioperative outcomes of patients. Several frailty assessment scores have been published since 2001, aiming to categorize elderly patients effectively, although a comprehensive geriatric assessment (CGA) remains the gold standard [31,32,33]. International recommendations, such as those from the SIGG, ESCP, and ACS NSQIP, advocate for the CGA in the preoperative evaluation of older colorectal surgery patients [34,35], and several lines of evidence support the role of the CGA in enhancing surgical outcomes for this category of patients, prompting initiatives such as the ongoing Swedish trial assessing the impact of the CGA on elderly patients with colorectal cancer [36]. In our center, a prospective study pilot called “MINDS”, focused on preoperative and postoperative geriatric assessments for patients over 75 scheduled for colorectal surgery, is recruiting with the aim of stratifying elderly patients according to frailty criteria. Thus, we can compare their outcomes with those of a control group that undergo surgery without frailty assessments.

## 5. Conclusions

The aim of this study was to evaluate the feasibility of colorectal resection surgery (for both benign and malignant diseases) in elderly patients (over 75 years) in terms of short-term postoperative outcomes and to evaluate the feasibility of novel techniques, such as the minimally invasive approach. According to our results, age does not seem to be a clear contraindication for oncological colorectal resection (with the same radical approach used in younger patients) or for complex colorectal resection due to complicated diverticular diseases. However, more advanced oncological stages and a greater complexity of diverticular disease require an experienced and dedicated surgical colorectal team. Even if our series supports the feasibility and safety of colorectal surgery in elderly patients via a novel technique, this category of patient needs multidisciplinary perioperative support to reduce the need for intensive care units (ICUs), reduce the higher incidence of minor complications and reduce the length of hospital stay, which will optimize the expectancy and quality of postoperative life.

## Figures and Tables

**Table 1:** Baseline characteristics.

Variables	EG (53 pts)	YG (95 pts)	*p* Value
Median age (interquartile range)	81 y (75–92)	62 y (35–74)	-
Gender (M:F)	27:26	51:44	0.749
Median BMI	25.46	24.8	0.393
(interquartile range)	(22.5–28.3)	(21.6–27.7)	
*ASA Classification*			
Stage 1	2 (3.8%)	14 (14.7%)	0.002
Stage 2	28 (52.8%)	65 (68.4%)	
Stage 3	20 (37.7%)	15 (15.8%)	
Stage 4	3 (5.7%)	1 (1.1%)	
Median CCI (Charlson Comorbidity Index)	7	3	0.001
(interquartile range)	(5–10)	(2–6)	
Indication for surgical treatment			
Benign disease	9 (17%)	58 (61.1%)	0.006
Malign disease	44 (83%)	37 (38.9%)	

**Table 2:** Baseline characteristics of colorectal cancer patients.

Variables	EG (44 pts)	YG (59 pts)	*p* Value
Complicated patter	24 (54.5%)	23 (39%)	0.117
Stenosis	16 (36.4%)	14 (23.3%)	
Occlusion	1 (2.3%)	6 (10%)	
Perforation	0	1 (1.7%)	
Anemia due to bleeding	9 (20.5%)	2 (3.3%)	
Tumor localization			0.491
Right colon	24 (54.5%)	24 (40.7%)	
Left colon	12 (27.3%)	24 (40.7%)	
Rectum	8 (18.2%)	11 (18.6%)	
pTNM			
Stage 1	17 (38.7%)	23 (39%)	0.972
Stage 2	14 (31.8%)	13 (22%)	0.264
Stage 3	9 (20.5%)	19 (32.2%)	0.185
Stage 4	4 (9%)	4 (6.8%)	0.66
Harvested lymph nodes			
Median (interquartile range)	20 (15–28)	22 (15–32)	0.662

**Table 3:** Baseline characteristics of patients who underwent surgery for complicated diverticular disease.

Variables	EG (9 pts)	YG (36 pts)	*p* Value
Gender (M/F)	1:8	16:20	0.070
Indication for surgical treatment			0.017
Stenosis	6 (66.7%)	7 (19.4%)	
Acute perforation/abscess	0	4 (11.1%)	
Chronic perforation/abscess	3 (33.3%)	5 (13.9%)	
Recurrent acute episodes	0	20 (55.5%)	

**Table 4:** Perioperative outcomes (* indicates a significant differences between two groups).

Surgical and Postoperative Characteristics	EG (53 pts)	YG (95 pts)	RR (in EG)	95% CI
Surgical approach				
(Minimally invasive vs. open)	49 (92.5%) vs. 4 (7.5%)	90 (94.7%) vs. 5 (5.3%)	0.98	(0.89–1.07)
Conversion to open approach	7 (14.3%)	7 (7.7%)	1.79	(0.66–4.84)
Systemic failure	2 (4.1%)	0	8.89	(0.43–181.79)
Oncological reasons	2 (4.1%)	5 (5.5%)	0.72	(0.14–3.57)
Anatomical reasons	3 (6.1%)	2 (2.2%)	2.69	(0.46–15.59)
Definitive stoma creation	4 (7.5%)	3 (3.1%)	2.39	(0.56–10.28)
Temporary ileostomy creation	3 (5.7%)	12 (12.6%)	0.45	(0.13–1.52)
Ileostomy closure at 2-month FUP	3/3 (100%)	8/12 (66.6%)	1.34	(0.78–2.3)
ICU admission	21 (39.6%)	14 (14.9%)	2.69 *	(1.5–4.84)
Postoperative complications	25 (47.5%)	29 (30.5%)		
Grade 1–2	22 (41.5%)	24 (25%)	1.64 *	(1.03–2.63)
Paralitic ileus with NGT positioning	10 (18.8%)	2 (2%)	8.96 *	(2.04–39.39)
Acute urinary retention	6 (11.3%)	2 (3.1%)	3.58	(0.93–13.75)
Wound infection	6 (11.3%)	1 (1%)	1.79	(0.61–5.28)
Grade 3–4	3 (5.7%)	6 (6.25%)	0.9	(0.23–3.44)
Length of hospital stay (days) Median (interquartile range)	6 (5–7)	5 (5–6)		

## Data Availability

Data are unavailable due to privacy restrictions.
